# The impact of a pharmacist-managed dosage form conversion service on ciprofloxacin usage at a major Canadian teaching hospital: a pre- and post-intervention study

**DOI:** 10.1186/1472-6963-5-48

**Published:** 2005-06-29

**Authors:** Bradley P Ho, Tim TY Lau, Robert M Balen, Terryn L Naumann, Peter J Jewesson

**Affiliations:** 1 Pharmaceutical Sciences Clinical Services Unit, Vancouver General Hospital, Vancouver Coastal Health, 855 West 12^th^. Avenue, Vancouver, BC, Canada, V5Z 1M9; 2 Faculty of Pharmaceutical Sciences, University of British Columbia, 2146 East Mall, Vancouver, BC, Canada, V6T 1Z3

## Abstract

**Background:**

Despite cost containment efforts, parenteral (IV) ciprofloxacin appears to be overutilized at Vancouver General Hospital. In November 2003, the Pharmacist-managed intravenous to oral (IV-PO) Dosage Form Conversion Service was implemented, enabling autonomous pharmacist-initiated dosage form conversion for ciprofloxacin. This study evaluates characteristics of ciprofloxacin use prior to and following implementation of this conversion service.

**Methods:**

This was a single-centre, two-phase (pre/post), unblinded study. Phase I occurred between November 12, 2002 and November 11, 2003 (365 days), and Phase II between November 12, 2003 and March 11, 2004 (120 days). All patients receiving ciprofloxacin IV during these periods were reviewed. The primary endpoint was IV:PO ciprofloxacin use ratio. Secondary endpoints were total number of ciprofloxacin doses, proportion of inappropriate IV ciprofloxacin doses, cost of therapy between phases, and estimated cost avoidance with the intervention.

**Results:**

Two hundred ciprofloxacin IV treatment courses were evaluated (100 per phase). The IV:PO ciprofloxacin use ratio was 3.03 (Phase I) vs. 3.48 (Phase II). Total number of doses and ratio of IV to total doses across phases were similar (p = 0.2830). IV-PO ciprofloxacin conversion occurred in 27/100 (27%) of IV courses in Phase I and 23/100 (23%) in Phase II. Proportion of inappropriate ciprofloxacin IV doses decreased between Phases I and II (244/521 (47%) vs. 201/554 (36%) (p = 0.0005), respectively). Furthermore, the proportion of pharmacist-preventable inappropriate ciprofloxacin IV doses was reduced between Phases I and II (114/244 (47%) vs. 65/201 (32%) (p = 0.0026). Proportional cost avoidance associated with total inappropriate IV use was $7,172/$16,517 (43%) (in Canadian dollars) in Phase I vs. $6,012/$17,919 (34%) in Phase II (p = 0.001). Similarly, proportional cost avoidance associated with pharmacist-preventable inappropriate IV doses was reduced from $3,367/$16,517 (20%) in Phase I to $1,975/$17,919 (11%) in Phase II (p = 0.001).

**Conclusion:**

While overall utilization of ciprofloxacin remained unchanged and the proportion of IV to total doses was stable during the study period, the proportion of inappropriate IV doses and its associated costs appear to have declined subsequent to implementation of a Pharmacist-managed IV-PO Dosage Form Conversion Service. Such a program may be a beneficial adjunct in facilitating appropriate and cost-effective usage of ciprofloxacin.

## Background

The annual drug expenditures at Vancouver Hospital are approximately $13 million (in Canadian dollars). At the Vancouver General Hospital (VGH) site, anti-infectives accounted for an expenditure of $3.39 million or 25% of the 2002–03 fiscal year total drug costs. Ciprofloxacin ranked third among all drugs by cost and was the second highest annual expenditure within the anti-infective drug class at $646,000. In addition, ciprofloxacin expenditures increased 5% from the previous year. Of the 39,147 ciprofloxacin doses administered in the 2002–03 fiscal year, 18,297 doses (47%) were given intravenously (IV).

Ciprofloxacin is a fluoroquinolone antibacterial that is primarily active against aerobic gram-negative bacterial infections [1]. It can be administered via the IV and oral (PO) routes. Concentrations similar to those achieved with the IV formulation are possible when administering ciprofloxacin orally, as it is highly bioavailable [2,3]. The daily drug cost (including material and labour costs for dispensing, preparation, and administration) of a typical ciprofloxacin 400 mg IV regimen administered twice daily is $72.62 [4]. Conversely, the daily drug cost of an equivalent ciprofloxacin 500 mg PO regimen given twice daily is $8.48, or 12% of the IV regimen [4].

In an effort to optimize use and minimize drug expenditures, ciprofloxacin has been designated a reserved antimicrobial drug (RAD) at our institution and has been included in the existing intravenous to oral (IV-PO) Step-down Program since 1992 [5]. Under this initiative, the use of the PO formulation of ciprofloxacin has been promoted at our institution through the means of newsletters [6], chart talkers and notes [7,8], and direct pharmacist-physician interactions. Other drugs included in this program are cefuroxime, cefixime, clindamycin, fluconazole, levofloxacin, and metronidazole.

Despite these cost containment efforts, there is some evidence to suggest that the IV formulation of these drugs may not be optimally utilized. Previous investigations by others and ourselves have shown that the IV formulation is often initiated when the PO formulation can be used [9-13]. Of equal importance, conversion to the PO formulation does not appear to be undertaken in a timely manner [6,10,11,13,14]. This results in unnecessary medication costs, IV drug administration expenses, and potential exposure to adverse events associated with IV therapy (e.g. pain at injection site, phlebitis, and line infections).

Various authors have described criteria-based medication dosage form conversion programs in the literature [8,10,11,13,15-24]. Some institutions have implemented modified IV-PO conversion programs in which pharmacists are given the authority and responsibility to change dosage forms in accordance with established criteria (i.e. clinical stability of the patient, ability to tolerate PO medications, and lack of drug interactions that may impair drug absorption from the gastrointestinal tract) [10,17,18,23,24]. The anticipated benefit of a pharmacist-managed conversion program is that delays in IV-PO conversion will be reduced if a pharmacist can avoid the requirement of conferring with the initiating physician before commencing a dosage form change. These services have demonstrated that cost savings can be achieved when pharmacists are directly responsible for changing the route of administration for selected medications [24].

In November 2003, the Pharmacist-managed IV-PO Dosage Form Conversion Service was approved at VGH by the Antibiotic Use Subcommittee (AUS), Drugs and Therapeutics Committee (D&TC), and the Medical Advisory Committee (MAC). Several antimicrobial agents were included in this service; namely, ciprofloxacin, clindamycin, co-trimoxazole, fluconazole, levofloxacin, metronidazole, acyclovir, ampicillin, cefazolin, cefuroxime, penicillin G, ceftriaxone, imipenem-cilastatin, cloxacillin, erythromycin, and ticarcillin-clavulanate [25].

Our hypothesis was that ciprofloxacin IV was overutilized at VGH and that a more cost-effective use of this dosage form was possible with the implementation of a Pharmacist-managed IV-PO Dosage Form Conversion Service. Accordingly, this study was conducted to assess the impact of this service on the relative utilization of the IV versus PO dosage form of ciprofloxacin. To our knowledge, there are no published reports involving an assessment of the impact of a Pharmacist-managed IV-PO Dosage Form Conversion Service on ciprofloxacin usage characteristics at a major Canadian teaching hospital.

## Methods

### Literature review

A literature search of the Medline, EMBASE and IPA databases, as well as a bibliographic review from the cited articles was performed to retrieve references pertaining to pharmacy-managed IV-PO conversion programs. Additional references were obtained through the St. Paul's Hospital and Lions' Gate Hospital pharmacy departments, as these local institutions had established pharmacist-managed conversion services [18,23]. The information collected was used to formulate a policy and procedure for the Pharmacist-managed IV-PO Conversion Service at our institution. This document was approved by the AUS, D&TC, and MAC, and a hospital-wide service was implemented.

### Study design

This was a single-centre, 2-phase (pre/post), unblinded study to assess the impact of a hospital-approved intervention aimed at improving the utilization of ciprofloxacin dosage forms. Phase I (365 days; November 12, 2002 to November 11, 2003) was designed to characterize ciprofloxacin usage patterns under the existing IV-PO Step-down Program. Phase II (120 days; November 12, 2003 to March 11, 2004) was designed to characterize the impact of the new Pharmacist-managed Dosage Form Conversion Service (implemented on November 12, 2003) on the relative utilization of the ciprofloxacin IV and PO dosage forms.

The primary endpoint was the relative utilization of IV and PO ciprofloxacin by dose (IV:PO ciprofloxacin use ratio). The secondary endpoints were the overall utilization of ciprofloxacin (by dose), the proportion of total IV doses considered to be inappropriate, and relative IV and PO total and treatment course acquisition costs between the two phases. Potential cost avoidance associated with the intervention was estimated from the data.

### Intervention

Prior to the implementation of the program, pharmacists were educated on the approved conversion service through in-house presentations. A newsletter was distributed to all medical staff detailing the program [25].

Decentralized clinical pharmacists on the medical wards were expected to conduct target drug report reviews 5 days per week to identify inpatients who had been prescribed ciprofloxacin IV. Health records were then reviewed, and patients assessed to determine if IV-PO conversion criteria were met. A patient was eligible for IV-PO dosage form conversion after 48 hours of IV therapy if he/she 1) continued to need an antibiotic; 2) was clinically stable; 3) was capable of tolerating the PO dosage form; and 4) had no factors present that would adversely affect PO bioavailability (e.g. gastrointestinal abnormalities or drug interactions). Pharmacists could consult the Infectious Diseases service or Infectious Diseases Pharmacist at anytime with any questions regarding IV-PO conversion eligibility.

For patients who met the conversion criteria, the pharmacist would write the order for the PO regimen in the Physician's Orders section of the health record. If the pharmacist wanted to convert the patient to PO ciprofloxacin prior to 48 hours of IV therapy, they would first confer with the physician. In collaboration with the healthcare team, the pharmacist would monitor the patient for clinical progress and medication tolerability, and could convert the patient back to IV therapy as required.

A randomly selected convenience sample of 200 ciprofloxacin IV treatment courses (100 treatment courses per phase) was considered to be adequate to determine the impact of this new intervention. These courses were identified using computerized pharmacy records and a random sample was undertaken using a computer-generated number list.

All patients who were ordered ciprofloxacin IV were included in the sample collection. Patients were excluded if they did not receive any doses of ciprofloxacin IV, if their ciprofloxacin IV treatment course did not occur within the pre-specified phase to which they were randomized, or if their charts were unavailable from the Health Records department at VGH as of July 14, 2004.

Charts were reviewed to gather demographic data, ciprofloxacin utilization information, inappropriate IV doses, and pharmacist-preventable IV doses of ciprofloxacin.

### Data collection and analysis

Data was collected by one investigator and entered into statistical analysis software (SPSS^© ^Version 11.0). Any treatment course that this investigator considered to have four or more inappropriately administered ciprofloxacin IV doses was reviewed in collaboration with the coordinating investigator to ensure accuracy of interpretation.

Inferential statistics were performed. A two-sample Student's t-test was used for parametric data, the Mann-Whitney test was used for non-parametric data, and the Fisher's Exact and Chi-square tests were used for proportional analyses.

### Definitions

For the purposes of this study, a ciprofloxacin IV dose was considered "inappropriate" when the patient met the criteria for use of the PO dosage form. A ciprofloxacin IV dose was considered "pharmacist-preventable" if the dose was administered when the patient met the criteria for use of the PO dosage form and the decentralized clinical pharmacist was considered to have had the opportunity to intervene (i.e. Monday to Friday between 08:00 and 16:00 hours, excluding statutory holidays). The inappropriate IV-PO ciprofloxacin acquisition cost was the differential cost between the IV and the PO dosage form at current contract prices multiplied by the number of inappropriate IV doses administered.

## Results

Two hundred and fifteen health records of patients who were prescribed ciprofloxacin IV during the study period were reviewed. Of these, seven patients were excluded, as the ciprofloxacin IV treatment courses were not completed within the pre-specified treatment phase. Six patients were excluded, as no IV ciprofloxacin doses were actually received. Health records were not accessible at the time of the study for the remaining two patients. Accordingly, 200 treatment courses for 200 patients (100 per phase) were included for analysis. This represented a 4% (100/2411 treatment courses) sampling rate for Phase I and a 10% (100/994 treatment courses) sampling rate for Phase II.

Patient demographics are presented in Table 1. Patients receiving ciprofloxacin IV were equally distributed by gender, typically in their sixth/seventh decade of life with an average duration of hospital stay of approximately two weeks. Treatment courses were initiated in both surgical and medical service areas for a wide variety of infectious indications. There were no significant differences between the two phases in terms of age, gender, renal function, length of stay, and medical service area to which the patients were assigned. Most patients (75% in Phase I, 78% in Phase II) received IV ciprofloxacin in combination with one or more antibiotics.

**Table 1:** Patient demographics

	**Phase I Nov. 12, 02 to Nov. 11, 03 (365 days)**	**Phase II Nov. 12, 03 to Mar. 11, 04 (120 days)**
No. of patients	100	100
No. of treatment courses	100	100
Age (yr), median (range)	57 (17–93)	63 (16–91)
Gender, N		
Male	45	50
SCr^1 ^(μmol/L), median (range)	84 (40–541)	89 (35–641)
Length of Stay (d), mean (range)	12 (1–84)	17 (1–165)

***Service Area, N***		
General Surgery	31	30
Medicine	22	11
Emergency	15	11
Intensive Care Unit	7	8
Urology	3	12
Other	22^2^	28^3^

***Indication, N***		
Off-label indications^4^	38	35
Intra-abdominal infection	18	15
Respiratory tract infection	15	16
Urinary tract infection	15	15
Other	14^5^	19^6^

^1^***Serum creatinine ***closest to start of ciprofloxacin IV treatment.

^2 ^***Other service areas***: Thoracic, Respiratory, Spine, Hematology/BMT, Transplant, Cardiothoracic Surgery, Neurosciences Intensive Care Unit, Cardiology, Day Bed Unit, Same Day Admit Unit, Pre-admission Clinic, Trauma Special Care Unit, Neurosciences, Family Practice, Orthopedics, Vascular, and Gynecology.

^3 ^***Other service areas***: Thoracic, Spine, Hematology/BMT, Transplant, Cardiology, Day Bed Unit, Same Day Admit Unit, Neurosciences, Family Practice, Vascular, Gynecology, Palliative Care, and Trauma.

^4^***Off-label indications ***refer to those not approved on the manufacturer's drug monograph.

^5^***Other indications***: Empiric therapy in febrile neutropenia, skin and soft tissue, and septicemia.

^6 ^***Other indications***: Empiric therapy in febrile neutropenia, skin and soft tissue, septicemia, and infectious diarrhea.

**Figure 1:**
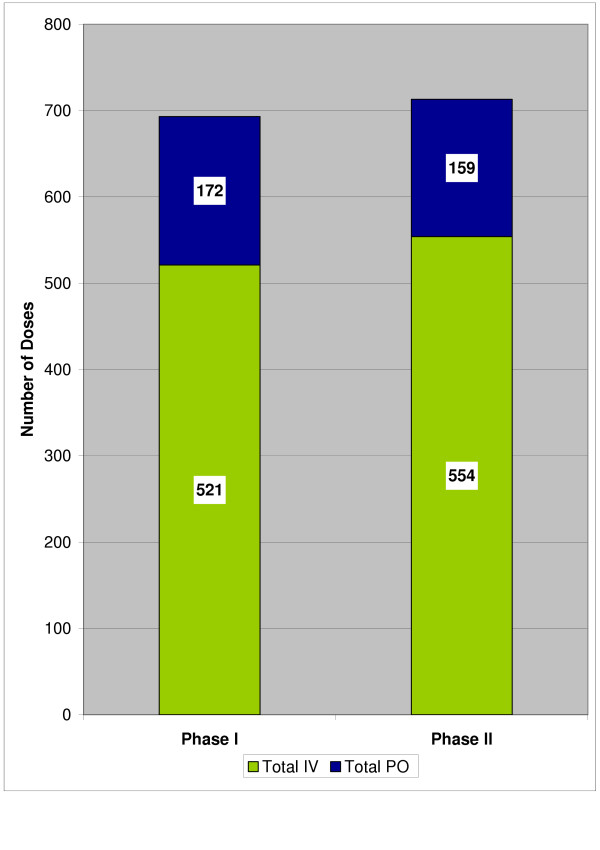
**Total number of ciprofloxacin doses**. p = 0.2830 for ratio of IV to total number of ciprofloxacin doses between phases.

Of the 200 ciprofloxacin IV courses reviewed, the total number of doses and the ratio of IV to total doses across phases were similar (p = 0.2830) (Figure 1). The IV:PO ciprofloxacin use ratio was 3.03 in Phase I vs. 3.48 in Phase II.

Ciprofloxacin treatment characteristics are described in Table 2. No significant differences were observed between the initial ciprofloxacin dosing strengths (p = 1.00), the initial dosing frequencies (p = 0.55), and the number of IV-PO conversions per treatment course (p = 0.73). IV-PO ciprofloxacin conversion occurred in 27/100 (27%) of IV treatment courses in Phase I and 23/100 (23%) of courses in Phase II (Table 2). The number of IV-PO conversions that were subsequently reversed to IV was 2 cases in Phase I and 3 cases in Phase II. Chart documentation of a pharmacist-initiated IV-PO conversion was recorded in 3/27 (11%) episodes in Phase I and 4/23 (17%) episodes in Phase II. There was no difference between phases with respect to the median number of ciprofloxacin doses administered and the median costs associated with each treatment course (Table 2).

**Table 2:** Ciprofloxacin treatment course characteristics

	**Phase I**	**Phase II**	**P value**
***Treatment regimen characteristics***			
*Dosing Strength*			1.00
200 mg IV	6	6	
400 mg IV	94	94	
*Dosing Frequency*			0.55
Once	17	22	
Once daily	5	3	
Twice Daily	78	75	
*IV to PO Conversion Rate (% by treatment course)*	27	23	0.73

Ciprofloxacin doses/treatment course, median (range)	5 (1–33)	5 (1–44)	0.55
IV, median (range)	3 (1–33)	4 (1–25)	0.29
Inappropriate IV, mean (range)	2.4 (0–26)	2.0 (0–9)	0.33
Inappropriate & pharmacist-preventable IV, mean (range)	1.1 (0–24)	0.6 (0–6)	0.14
PO, mean (range)	1.7 (0–26)	1.6 (0–26)	0.83

***Treatment course acquisition costs ($)***			
Ciprofloxacin, median (range)	99 (17–1089)	132 (17–825)	0.32
IV, median (range)	99 (17–1089)	132 (17–825)	0.28
Inappropriate IV, mean (range)	72 (0–790)	60 (0–274)	0.39
Inappropriate & pharmacist-preventable IV, mean (range)	34 (0–729)	20 (0–182)	0.17

**Figure 2:**
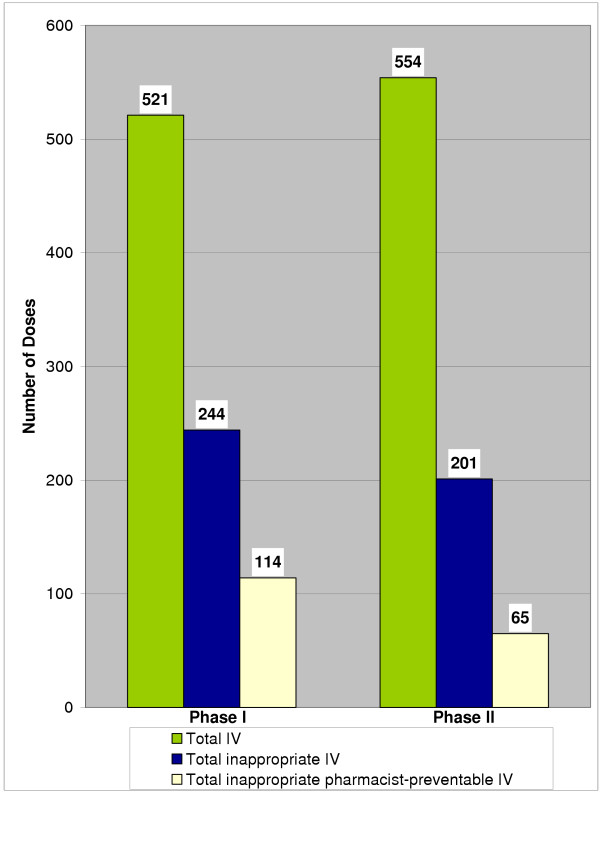
**Number of total, inappropriate, and pharmacist-preventable inappropriate IV ciprofloxacin doses. **p = 0.0005 for difference in the proportions of inappropriate IV ciprofloxacin doses between phases. p = 0.0026 for difference in the proportions of pharmacist-preventable inappropriate IV ciprofloxacin doses between phases.

For those patients who met the criteria for the use of an oral dosage form, 59/100 (59%) received one or more inappropriate doses of ciprofloxacin IV in Phase I compared to 61/100 (61%) in Phase II. There was a significant decrease in the proportion of inappropriate ciprofloxacin IV doses between phases (244/521 (47%) in Phase I vs. 201/554 (36%) in Phase II (p = 0.0005) (Figure 2). Furthermore, there was a significant reduction in the proportion of pharmacist-preventable inappropriate ciprofloxacin IV doses between Phase I and Phase II (114/244 (47%) vs. 65/201 (32%) (p = 0.0026) (Figure 2).

**Figure 3:**
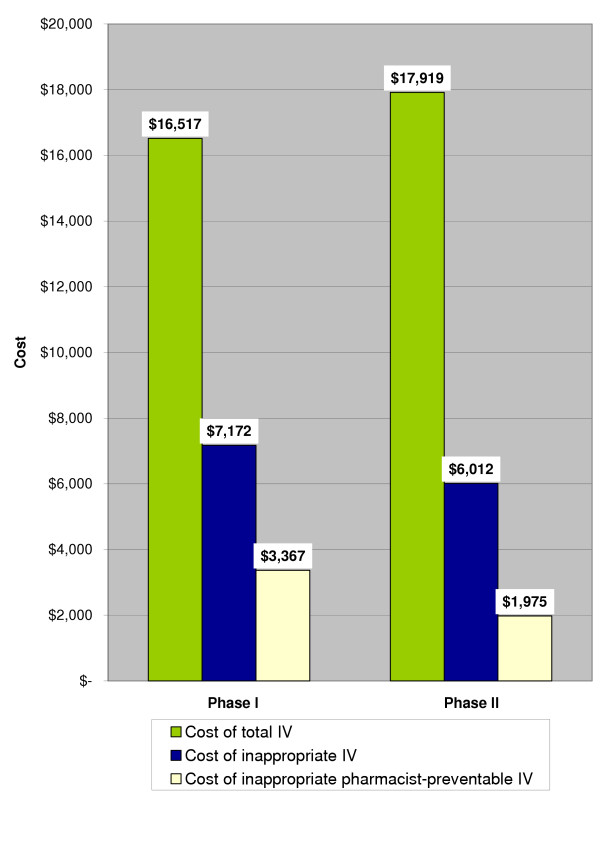
**Costs associated with total, inappropriate, and pharmacist-preventable inappropriate IV ciprofloxacin doses. **p = 0.001 for difference in potential cost avoidance of inappropriate IV ciprofloxacin doses between phases. p = 0.001 for difference in potential cost avoidance of pharmacist-preventable inappropriate IV ciprofloxacin doses between phases.

The total cost of IV and PO ciprofloxacin for the treatment courses reviewed was $16,993 in Phase I and $18,332 in Phase II. Ciprofloxacin IV accounted for $16,517 (97%) of total ciprofloxacin costs in Phase I and $17,919 (98%) of these costs in Phase II (Figure 3). The proportional cost avoidance associated with inappropriate use of IV ciprofloxacin was $7,172/$16,517 (43%) in Phase I compared to $6,012/$17,919 (34%) in Phase II (p = 0.001). The proportional pharmacist-preventable cost avoidance associated with inappropriate IV ciprofloxacin use was reduced from $3,367/$16,517 (20%) in Phase I to $1,975/$17,919 (11%) in Phase II (p = 0.001).

## Discussion

The purpose of this study was to assess the relative utilization of the IV and PO dosage form of ciprofloxacin subsequent to the implementation of the Pharmacist-managed Dosage Form Conversion Service. We did not aim to assess the appropriateness of ciprofloxacin usage for specific indications.

Overall, the IV:PO ratio of ciprofloxacin usage remained similar between the two phases, and the total number of ciprofloxacin doses did not change significantly. Initially, it was anticipated that implementation of the conversion service would reduce the IV:PO ratio, however, there were numerous variables that may have affected this endpoint. We were also interested in evaluating whether the number of inappropriate IV doses and pharmacist-preventable inappropriate IV doses could be reduced. Our results showed a 23% relative reduction in the proportion of inappropriate ciprofloxacin IV doses and a 32% relative reduction in the incidence of pharmacist-preventable inappropriate ciprofloxacin IV doses subsequent to the intervention.

A possible explanation for the decline in the inappropriate and pharmacist-preventable inappropriate ciprofloxacin IV doses was the drive to reduce hospital expenditures at our institution at the time this program was implemented. There was an increased awareness and emphasis for cost-effective prescribing. The conversion service was an adjunct to the cost savings initiatives and was readily adapted to our established practice.

VGH has had a pre-existing IV-PO Step-Down Program since 1992. In a previous study at our institution in 1992, the rate of patients eligible for IV-PO conversion was 52%, which is similar to the 59% observed in Phase I of our present study [6]. The rate of IV-PO conversions in 1992 was also comparable to our baseline in Phase I (34% vs. 27%, respectively). This suggests that the interventions of our IV-PO Step-down Program have remained relatively constant since 1992. With an effective IV-PO Step-down Program in place, it is possible that the magnitude of change associated with the implementation of the conversion service may have been blunted.

Pharmacist-initiated IV-PO conversion was documented in the health record in 4 cases in Phase I and 3 cases in Phase II. This low incidence of documentation may be attributed to the activities of the decentralized clinical pharmacists who attend patient care rounds and interact directly with physicians, so that orders are written during rounds for IV-PO conversion. Greater awareness of other health care professionals on the bioavailability of PO ciprofloxacin may also have resulted in the earlier usage of the PO dosage form.

Total costs of ciprofloxacin therapy were similar between the two phases. This was expected, as the conversion service would not alter the indications for ciprofloxacin use. However, the costs associated with inappropriate ciprofloxacin IV therapy and pharmacist-preventable inappropriate ciprofloxacin IV therapy declined from Phase I to II, which may be attributed to the increased interventions of the clinical pharmacists and the improved awareness for PO therapy post intervention.

Following implementation of the conversion service, 12% (65/554) of ciprofloxacin doses deemed inappropriate and preventable by pharmacists were still administered. Ideally, all of these doses should have been avoided. One full working day (excluding weekends and statutory holidays) was allotted as the time required for clinical pharmacists to assess these patients. This delay in IV-PO conversion may be explained in part by having our data collection period immediately after the introduction of the new service, as pharmacists may not yet have been comfortable exercising a dosage form conversion autonomously. The retrospective assessment for appropriateness by the investigator may also differ from that of the clinical pharmacist. It would be beneficial to obtain an internal assessment to discover the barriers associated with the program. Of course, it would be preferable to educate the medical staff to initiate PO regimens where indicated and avoid the use of the IV formulation.

Several limitations exist with this study. The retrospective, pre/post, unblinded design precludes the formulation of any direct causal relationships between the implementation of the conversion service and the subsequent reduction in inappropriate ciprofloxacin IV doses. The sample size of convenience may not have achieved the power required to detect a difference. In addition, the sampling rate was relatively low at 4.1% (100/2411 ciprofloxacin IV courses) in Phase I and 10.1% (100/994) in Phase II, and thus may not truly represent the characteristics of our population. Time restrictions resulted in differences in sampling periods between Phase I (365 days) and Phase II (120 days), which may affect the representation of ciprofloxacin IV treatment courses throughout the year. However, this should not directly influence the proportion of inappropriate and pharmacist-preventable inappropriate ciprofloxacin IV treatment doses. The assessment of this service was over a relatively short period, and so may not truly reflect the long-term impact of this program.

As with any unblinded study of this type, the potential for investigator assessment bias existed. As the evaluation of dose appropriateness was undertaken sequentially across phases, potential bias may have been introduced as the investigator gained experience during the process. To minimize this bias, charts were reviewed by a senior investigator when greater than four inappropriate and pharmacist-preventable inappropriate ciprofloxacin IV doses were identified by the junior investigator. A 100% concordance existed between the assessments made by the junior and senior investigators.

In the context of drug use optimization and cost minimization, implementation of a Pharmacist-managed IV-PO Dosage Form Conversion Service may be used to facilitate appropriate, cost-effective therapy. This service can be used in conjunction with other established methods including newsletters [6], chart talkers, notes [7,8], and direct pharmacist-physician interactions. To further promote antimicrobial use appropriateness, strategies aimed at affecting prescribing behaviour may be employed. These include individual physician prescribing feedback, multidisciplinary inservices in collaboration with infectious diseases physicians, and prescriber education through academic detailing.

## Conclusion

In summary, the overall utilization of ciprofloxacin seems to have remained unchanged and the proportion of IV to total doses appears stable. However, the proportion of inappropriate IV doses and its associated costs appear to have declined subsequent to the implementation of a Pharmacist-managed IV-PO Dosage Form Conversion Service. Such a program may be a beneficial adjunct in facilitating appropriate and cost-effective usage of ciprofloxacin.

## Competing interests

The author(s) declare that they have no competing interests.

## Authors' contributions

BPH participated in the design of the study, performed data collection and analyses, and drafted the manuscript. TTYL participated in the design and coordination of the study, performed data and statistical analyses, and drafted and revised the manuscript. RMB participated in the design of the study, developed the analytical database, and revised the manuscript. TLN participated in the design of the study and revision of the manuscript. PJJ conceived the study, participated in its design, performed data and statistical analyses, and drafted and revised the manuscript. All authors read and approved the final manuscript.

## Pre-publication history

The pre-publication history for this paper can be accessed here:


